# Maternal Androgens Increase Sibling Aggression, Dominance, and Competitive Ability in the Siblicidal Black-Legged Kittiwake (*Rissa tridactyla*)

**DOI:** 10.1371/journal.pone.0047763

**Published:** 2012-10-24

**Authors:** Martina S. Müller, Yvonne Roelofs, Kjell Einar Erikstad, Ton G. G. Groothuis

**Affiliations:** 1 Behavioural Biology, Centre of Behavioural Neurosciences, University of Groningen, Groningen, The Netherlands; 2 Department of Arctic Ecology, Norwegian Institute for Nature, Tromsø, Norway; Max Planck Institute for Evolutionary Anthropology, Germany

## Abstract

Animals and plants routinely produce more offspring than they can afford to rear. Mothers can favour certain young by conferring on them competitive advantages such as a leading position in the birth sequence, more resources or hormones. Avian mothers create hatching asynchrony within a clutch and at the same time bestow the eggs with different concentrations of androgens that may enhance or counteract the competitive advantage experienced by early-hatching “core” young. In siblicidal birds, core young assume a dominant social position in the nest due to their size advantage and when threatened with starvation fatally attack subdominant later-hatching “marginal” young. A role for maternal androgens in siblicidal aggression has frequently been suggested but never tested. We studied this in the facultatively siblicidal black-headed kittiwake. We found that marginal eggs contain higher instead of lower concentrations of androgens than core eggs. Surprisingly, exposure to experimentally elevated yolk androgens increased sibling aggression and dominance, even though in nature marginal eggs never produce dominant chicks. We propose the “adoption facilitation hypothesis” to explain this paradox. This cliff-nesting colonial species has a high adoption rate: ejected marginal kittiwake chicks frequently fall into other nests containing chicks of similar or smaller size and exposure to yolk androgens might help them integrate themselves into a foster nest.

## Introduction

With each reproductive attempt, organisms face the critical decision of how many progeny to produce. According to life history theory, their choices reflect an optimized trade-off between offspring quality and offspring quantity [1]–[3] as well as contributions of current versus future reproductive success to parental fitness [4], [5]. These trade-offs arise due to the costs of producing offspring and in many species, progeny number becomes limited by substantial investment into costly parental care during the early postnatal life of offspring [6]. The capacity of parents to deliver the requisite care to young depends heavily on resource availability during the reproductive season, which they are often unable to predict accurately [1], [7]. Animals as well as plants chronically overproduce offspring but appear to have evolved mechanisms to adjust their broods secondarily to a more modest number when the number of viable progeny exceeds what parents can afford to rear [8]. Mothers can either cull their brood directly via cannibalism (e.g. rodents [9], amphipods [10], fish [11]) or they can facilitate sibling rivalry-mediated brood reduction by conferring phenotypic handicaps or advantages on certain young (e.g. pronghorn embryos [12], seeds of *Dalbergia sissoo* [13], canary eggs [14]) so that competitively superior siblings can eliminate competitively inferior siblings when resources are scarce [15].

The role of maternal effects in modulating fatal sibling rivalry has been studied most extensively in birds, perhaps in part due to the conspicuous disadvantage imposed by many avian mothers on parts of their broods via hatching asynchrony [16]. Hatching asynchrony occurs when mothers begin incubating eggs before they finish laying the entire clutch which initiates early onset of development in the already-laid eggs (core eggs, *sensu* [17], [6]) and results in delayed development and hatching in later-laid eggs (marginal eggs, *sensu* [17], [6]). Asynchronous hatching causes chicks from marginal eggs (marginal chicks) to be smaller and competitively inferior and therefore to have much lower survival than their core siblings [18], [16]. In many avian species, marginal young die of starvation under poor food conditions when they repeatedly fail to receive food delivered by parents because of their relatively smaller size and retarded motor development (scramble competition [15]). Siblicidal species constitute a special case in which age and size asymmetries within broods translate into clear dominance hierarchies, and subordinate marginal young die primarily because dominant core siblings attack them, resulting in fatal wounds or exile from the nest [19], [6].

While within-brood size asymmetries are caused predominantly by hatching asynchrony [15], mothers may further fine-tune competitive asymmetries by allocating more androgens to certain eggs. Avian mothers deposit high concentrations of androgens into egg yolks that vary in systematic patterns over the laying sequence (for reviews see [20]–[22]). Exposure to elevated yolk androgens often promotes faster pre- and post-natal development in young, more begging to parents for food and increased territorial aggression (reviewed in [20]–[22]). Increasing and decreasing patterns of yolk androgen allocation over the laying sequence might be an adaptation to promote brood survival and brood reduction strategies respectively by compensating or enhancing within-brood size asymmetries caused by asynchronous hatching [14], [23], [20].

However, the function of yolk androgen exposure in the context of sibling competition has only been studied in species in which brood reduction occurs via scramble competition for food (reviewed in [22]), and has never been studied in a species in which broods are reduced by core siblings using overt aggressive behaviour against their marginal siblings. Following the publication of a very frequently-cited study reporting that total yolk androgens decrease over the laying sequence in the facultatively siblicidal cattle egret [24], numerous papers implied that relatively higher maternal androgens in core eggs might aid siblicidal brood reduction of marginal young by increasing aggression and/or dominance in the core young. Since then, variable patterns of yolk androgen deposition have been reported for other siblicidal species: yolk androgens increase over the laying sequence in black-legged kittiwake [25], in Australian pelicans (G. Johnston, unpublished data), and in blue-footed boobies in poor breeding conditions [26]; yolk androgens show no significant change over the laying sequence in brown and blue-footed boobies [27]; and yolk androgens decrease over the laying sequence in blue-footed boobies in good breeding conditions [26]. Yolk androgen exposure increases territorial aggression directed toward unrelated young in non-siblicidal gulls, but not to siblings [28], and its effects on siblicidal aggression in siblicidal species are still not known.

We investigated the function of within-clutch variation of yolk androgens for sibling competition and brood reduction in the facultatively siblicidal black-legged kittiwake (*Rissa tridactyla*), which lays a modal clutch size of two eggs [29]. When food is scarce, the first-hatching core chick eliminates the second-hatching marginal chick via aggressive attacks that cause the marginal chick to fall out of the nest [30]. But in years with sufficient food availability, core chicks tolerate the presence of the marginal chick, although a clear dominance hierarchy exists in which the subdominant chicks regularly performs submissive displays [30], [31].

In our study, we measured yolk androgen concentrations from first- and second-laid eggs to investigate whether yolk androgens increase over the laying sequence in our kittiwake population. Then we manipulated androgen concentrations in kittiwake eggs and created artificial sibling pairs matched for age and weight, containing one chick exposed to a high androgen level and one chick exposed to a lower yolk androgen level to determine whether exposure to yolk androgens enhances competitive ability in chicks and might therefore provide a mechanism for mothers to adaptively fine-tune competitive hierarchies within broods. If yolk androgens increase over the laying sequence, we hypothesize that mothers intend yolk androgens to compensate marginal young and should therefore enhance begging behaviour, speed up growth and development, but have no influence on aggression. If yolk androgens decrease over the laying sequence, we hypothesize that yolk androgens enhance aggressive behaviour towards the sibling so that mothers facilitate brood reduction by conferring a competitive advantage on core young via increased deposition of maternal androgens in the first egg.

## Materials and Methods

### Study Colony and Species

All eggs used in this study came from a large, dense black-legged kittiwake colony on the Ekkerøy peninsula in Norway. Approximately 20,000 pairs of kittiwakes breed on the south-oriented cliff that rises steeply 40–50 meters from the Varanger fjord and stretches ca. 1 km along the coast. Kittiwakes lay a modal clutch size of two eggs [32] with an average laying interval of 2.5 days between eggs [32]. In this species, the second chick hatches ca. 1.3 days after the first egg [30], [32].

### Ethics Statement

Egg collection was licensed by the Directoratet for Naturforvaltning in Tromsø, Norway. The experiment was executed according to a protocol approved by the Animal Experimentation Committee of the University of Groningen under license DEC 3543.

In the evenings, chicks from dyads that showed aggression were separated in cages by a barrier. In the cages in which we hadn’t observed aggression, we placed small wooden nest boxes into the cages so that submissive chicks could find refuge in case the dominant chick became aggressive.

Kittiwake chicks show two types of aggressive behaviour: low and high intensity aggression. Low intensity pecks serve to enforce a dominance hierarchy and elicit submissive postures from the subdominant sibling. Often dominant chicks lunge towards their sibling but their attempts to peck do not even result in contact with the sibling and represent more of a threat display. The second type of aggression is the escalated violence that precedes siblicide and is comprised of frequent intensive pecks. Over the short term this does not injure chicks although subdominant chicks move away to escape attacks. In kittiwake colonies, chicks occupy relatively small nests built on the sides of cliffs and siblicide occurs when aggressive dominant chicks attack subdominant chicks and push chicks out of the nest. Chicks lack the weaponry and strength to inflict serious injuries on their siblings. In our experiment, the aggression we observed were primarily the dominance pecks, and we only rarely observed escalated aggression. In the few cases in which aggression escalated to the intensity that precedes siblicide in a natural setting, chicks were immediately separated. We recorded presence/absence of aggression only before meals (ca. every 5 hours) to increase statistical independence of consecutive records of aggression. But we monitored chicks more frequently (ca. every 2 hours). Only occasionally did chicks begin to show intense aggression already well before meal times and in those cases they were separated.

A few chicks died from developmental problems at hatching and a few chicks died from unknown causes (we suspect a nutrient lacking from their diet). We undertook several actions to counteract the latter by contacting several experts and trying to optimize food with daily fresh fish for example (as this is their natural diet).

Most chicks were euthanized, but 6 chicks were raised in outdoor aviaries to adulthood, so that we could fine-tune the composition of their diet for future experiments with this species. After 4 months in the outdoor aviaries, these birds were also euthanized. Euthanizing the birds was mandated by the governmental inspector for ethical issues in animal experiments. We euthanized the chicks via lethal Pentobarbital injection as specified by animal welfare guidelines at our university.

### Egg Collection, Hormone Measurement, and Injection

In May 2009, we collected 14 freshly-laid eggs (seven first eggs, seven second eggs, all coming from 7 clutches) from the kittiwake colony in Ekkerøy. Empty nests were marked and checked every day for new eggs. After eggs were collected, yolks were immediately separated from albumin and stored at −20 degrees C. Yolks were later thawed, weighed, and diluted in 1 mL filtered, deionized water (Milli-Q) per gram of yolk. We extracted testosterone and androstenedione from approximately 300 mg of diluted yolk sample using an extraction and radioimmunoassay protocol described in [14].

**Figure 1:**
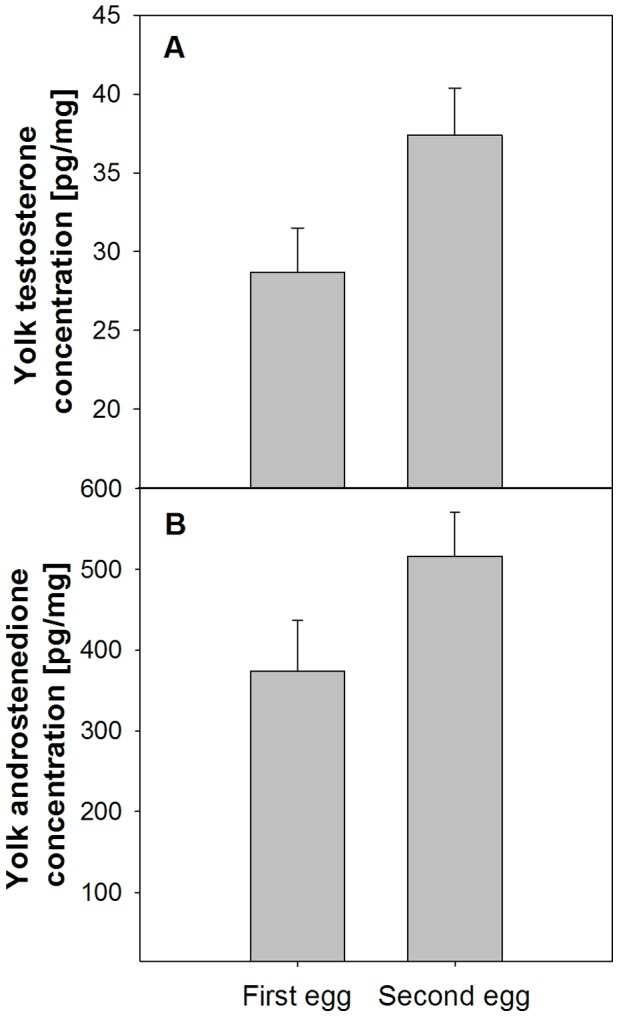
Means and standard errors of yolk testosterone (A) and androstenedione (B) concentrations in first and second kittiwake eggs.

In mid-May 2010, we monitored 176 nests in the Ekkerøy colony daily and collected freshly-laid first eggs only. Eggs were stored at ca. 12 degrees C for 1–5 days (below physiological zero) and then were transported back to the Netherlands and injected the following day. We injected 40 eggs with 50 µl of vehicle (sterile sesame oil) and 40 eggs with 50 µl of androgen solution of the same oil containing 0.153 µg testosterone and 2.695 µg androstenedione (Sigma, Germany). This amount of androgen represents the average difference in total testosterone and androstenedione between first and second-laid black-legged kittiwake eggs (testosterone levels from [25], androstenedione from [33], mean yolk size: 12.92 g, standard error: 0.215 g from [34]). Our androgen injections elevated androgen concentrations in eggs to a mean level that fell within one standard error of the mean concentrations we measured in second-laid eggs from our population (see results and Fig. 1A and 1B) We injected laterally-held eggs from an off-center point on the shell and inserted the needle at a 45 degree angle so that it would penetrate the yolk without damaging the blastocyst. Injections were made with a 25 G needle and the hole in the shell was closed with a tiny square of artificial skin (Hansaplast). We then placed all eggs into one incubator at 37.5 degrees C and 50% humidity until they hatched (ca. 26 days).

### Experimental Design

After all chicks hatched, we created artificial sibling dyads of age- and size-matched androgen and control chicks (mean age at pairing 29.5 hours, ranging from 0 to 51 hours old) and housed each dyad in individual cages with constant light mimicking the 24 hours of daylight they would experience in an arctic Norwegian summer. The experiment room was lit with fluorescent bulbs that meet the standards for animal housing facilities in the Netherlands. In addition, each cage was lit with a 25-Watt bulb during the first week of the experiment to provide warmth for very young chicks. Light intensity did not change over the course of the day. Chicks were fed a mixture of frozen cod and fresh European anchovy and smelt 5 times per day, and on day 20 of the experiment we began supplementing their diet with rehydrated trout pellets. Chicks remained in the same dyad for several days at a time (mean = 7.04 days, s.e. = 0.67) and then were swapped into new dyads because each new sibling combination presented a new opportunity to test the effect of yolk androgen exposure on dominance. We created 52 unique chick dyads in total (number of chicks experiencing at least one dyad: n = 39; at least 2 dyads: n = 32, at least 3 dyads: n = 20, at least 4 dyads: n = 12; 5 dyads: n = 1). Because several times during the experiment androgen chicks outnumbered control chicks, we occasionally created sibling dyads containing two androgen chicks. Even though those dyads did not allow us to test effects of elevated androgen exposure, we were able to include them in analyses investigating winner-loser effects of dominance status across consecutive dyads. The experiment lasted 28 days.

### Growth and Development

After hatching began, incubators were checked every 3 hours. We recorded hatching time and hatching success. We measured body mass and bill length of all chicks at hatching and at 10 additional time points thereafter (days 0, 2, 4, 6, 8, 14, 16, 18, 20 and 25 of the experiment). Bill length correlates linearly with tarsus in kittiwake chicks (Müller, unpublished data), indicating that it is a good measure of structural body size, but it has higher repeatability than tarsus measurements. We also recorded age and treatment of any deaths that occurred during the experiment.

### Behaviour

We scan-sampled aggressive and submissive behaviour in all dyads at sampling points spaced several hours apart (n = 48 sampling points, range in intervals between sampling: 1.9–74 hrs, median = 7 hrs). After entering the experiment room we noted which chicks were showing aggression toward their sibling, via aggressive pecks on the body, neck and head of the victim, and which chicks were showing submissive behaviour characterized by tucking the bill under the body and exposing the black band of down across the nape [29]. We also recorded whether chicks performed begging vocalizations during many of the same sampling points (n = 21 sampling points, range in intervals between sampling: 2.4–93 hrs, median = 8.6 hrs). All of these behaviours were registered as binary events: present or absent during our observation.

We performed begging tests on day 3 of the experiment (average chick age: 3.82 days, s.e. = 0.10), 4 hours after their 11 am feeding. Each chick was placed alone into an empty cage and filmed for 120 seconds. During the time intervals of 30–60 seconds and 90–120 seconds, chicks were presented with forceps holding a small morsel of fish as was standard practice during hand-feeding. To trigger regurgitation of food, kittiwake chicks thrust upward toward the parent’s bill and peck at it, much like in gull species [29] and in our experiment they performed a similar behaviour toward the forceps during feedings. Begging behaviour was later scored from videos by an observer blind to the chicks’ treatment and included the number of pecks at the forceps and the number of thrusts toward the forceps and toward the general direction of the observer. Begging vocalizations were difficult to distinguish reliably between chicks in the videos and were not analysed.

We also performed standardized sibling competition tests on the same day after all chicks were returned to their own cages together with their current sibling. The aim was to determine feeding order in a competitive setting. An observer blind to the chicks’ identities presented chicks with 10–15 morsels of fish and scored which chick ate which morsel in the sequence they were delivered.

### Statistical Analyses

All statistical analyses were performed in the R statistical computing environment [35]. Differences in yolk androgens between first and second eggs of the same clutches were tested using paired t-tests. We tested effects of treatment on hatching success and chick mortality using 2×2 Fisher Exact tests. We performed an unpaired Wilcoxon rank sum tests to test effects of treatment on hatching time, hatchling size, and age of chick mortality.

All measurements of chicks were taken before they approached asymptotic body mass, so mass and bill length increased linearly with age, which we confirmed via visual inspection of the data. Growth data were, therefore, analysed using linear mixed effects regression (nlme package [36]) with treatment as a fixed factor, age as a covariate and individual as a random factor.

Since aggression, submission and begging vocalizations were registered as binary variables, we analyzed these data using mixed effects binomial regression (lme4 package [37]). In all analyses testing effects of treatment on behaviour, we included only dyads that contained one chick from each treatment group and excluded the dyads containing two androgen chicks. In these models we included treatment as a fixed factor and chick age, residual body mass and mass differences between siblings as covariates. To compute residual body mass we regressed body mass on bill length for each chick, and used the model residuals as a proxy for the energy reserves of each chick. Mass differences between siblings were calculated for each chick as half of the mass difference between siblings: the larger chick was given a positive value, the smaller chick was given a negative value. We also tested for an effect of relative dominance on frequency of bouts of begging vocalizations, by computing half of the difference in dominance score (see below) between two siblings: the more dominant chick was given a positive value, the less dominant chick was given a negative value. In this model we included relative dominance and residual body mass as covariates. In all of the analyses of binary behavioural data, we included nest as a random factor because we were comparing chicks of the same dyad, and also chick because we used repeated measurements of the same chicks. Details about sample sizes are provided in the results section.

In certain dyads, chicks appeared to establish a dominance hierarchy more quickly and effectively than in other dyads. To determine whether elevated maternal androgen exposure produces more distinct dominance, we calculated a dominance score by dividing the frequency of observed aggression by the sum of the aggressive and submissive behaviour shown by the chick. Then we compared these dominance scores between both siblings in a dyad and identified the sibling with the larger dominance score as the dominant chick in the dyad. We used a linear mixed model (including only dominant chicks) to test whether dominant androgen chicks had a higher dominance score relative to their sibling than did dominant control chicks, by using dominance score as the dependent variable and treatment as fixed factor. In these models we included only chicks coming from dyads that contained one androgen chick and one control chick. We excluded dyads in which neither chick showed aggression or submission because it was not possible to identify a dominant individual. In these linear mixed models, we included chick as a random factor to correct for repeated observations of the same dominant chicks in different dyads.

To test whether previous dominance status determines dominance status when paired with a new sibling, we performed a binomial mixed model in which we used previous dominance status (1 =  dominant, 0 =  subdominant) as a predictor for dominance status in the subsequent dyad. The analysis included only dominance status from individuals in which we were able to calculate the dominance score for the current and subsequent dyad (i.e. in both dyads, we observed at least some aggression or submission). This data set included dyads including androgen and control chicks but also dyads including two androgen chicks. In these models, we included dyad and chick as random factors.

We analysed behavioural data from the begging tests using mixed effects Poisson regressions and in addition to the random factor of cage, we included an individual-level random effect that corrects overdispersion in the Poisson response variable [38].

We performed linear mixed effects regressions to test whether androgen chicks took the first meals in a sibling competition test faster than control chicks did. Each competition test included a series of sequential trials (10 to15) which exceeds the number of fish morsels required to satiate a chick by ca. 40% (personal observation). We used trial number as the response variable in our models, and included cage as a random factor to correct for the fact that each competition test on the sibling pairs contained several trials. The predictor in the first model was the treatment of the winning chick and in the second model the relative dominance of the winning chick. The direction of the relationship indicates which chick fed to satiety first. For example a negative relationship would indicate that testosterone chicks (coded as 1) won more of the early trials (lower numbers) and fewer of the later trials (higher numbers) than did control chicks (coded as 0).

## Results

### Yolk Androgens

Second-laid eggs contained substantially higher concentrations of testosterone (t = 5.451, df = 6, p = 0.0016) and androstenedione (t = 5.936, df = 6, p = 0.001) than did first-laid eggs (Fig. 1a, 1b; see “Statistical analyses” in Materials and Methods for details about statistical tests).

### Development, Growth and Survival

Hatching success did not differ between the two treatments (22 out of 40 androgen-injected eggs hatched, 17 out of 40 control-injected eggs hatched, p = 0.371). Androgen-injected eggs did not hatch significantly earlier than control-injected eggs (W = 192, p = 0.898). Hatchlings coming from androgen-injected eggs did not differ in mass (W = 237, p = 0.161) or bill length (W = 179, p = 0.832) from hatchlings coming from control-injected eggs.

We found no effect of treatment on mass (treatment: b = 0.371, s.e. = 4.458, df = 37, p = 0.931; age: b = 4.223, s.e. = 0.184, df = 251, p<0.001) or on bill growth (treatment: b = 0.768, s.e. = 0.625, df = 37, p = 0.227; age: b = 0.999, s.e. = 0.016, df = 251, p<0.001). Androgen chicks and control chicks showed similar mortality rates (Fisher Exact test: p = 1.000) and did not differ in the age at which they died (W = 51, p = 0.160, 11 controls vs. 14 androgen chicks).

### Aggression

**Figure 2:**
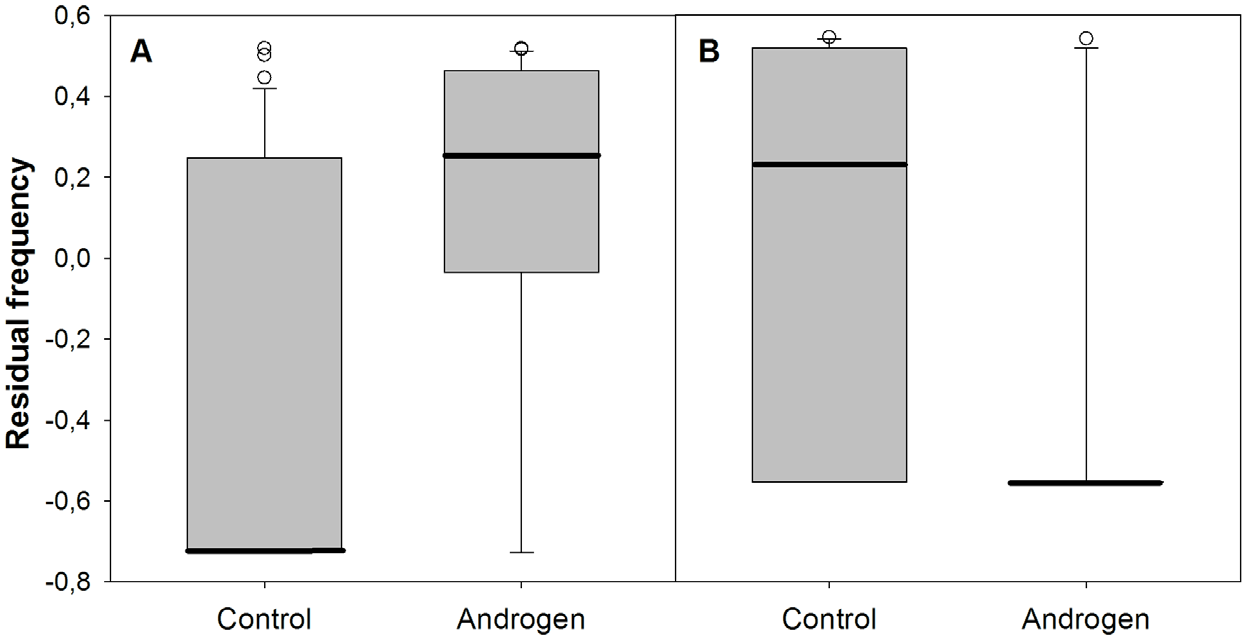
Residual frequency of bouts of aggression (A) and submission (B) in chicks coming from control and androgen-treated eggs. Residuals in figures produced by Poisson mixed effects regressions including individual and dyad as random factors.

Chicks from androgen-injected eggs showed more frequent bouts of aggression than did chicks from control-injected eggs (b = 2.128, s.e. = 0.516, p<0.001, n = 836 observations, 52 dyads, 39 individuals, Fig. 2a). Aggression decreased significantly with age (b = −0.109, s.e. = 0.031, p<0.001) but showed no relationship with residual body mass (b = 1.030, s.e. = 1.232, p = 0.403) or mass difference between siblings (b = 0.017, s.e. = 0.238, p = 0.491).

In an identical model in which we replaced mass asymmetry with bill length asymmetry we found qualitatively the same results: chicks from androgen-injected eggs showed more frequent aggression (b = 2.063, s.e. = 0.513, p<0.001), and aggression decreased with age (b = −0.108, s.e. = 0.031, p<0.001) but was not significantly predicted by residual mass (b = 0.667, s.e. = 1.131, p = 0.555) or bill length difference between siblings (b = 0.051, s.e. = 0.201, p = 0.807).

### Submission

We found a strong negative effect of androgen-injection on submissive behaviour (b = −2.176, s.e. = 0.579, p<0.001, n = 836 observations, 52 dyads, 39 individuals, Fig. 2b). In this model we found no significant association of submissive behaviour with age (b = −0.030, s.e. = 0.031, p = 0.351), residual body mass (b = −2.013, s.e. = 1.448, p = 0.164), or mass asymmetry (b = −0.021, s.e. = 0.028, p = 0.464).

In an identical model in which we replaced mass asymmetry with bill asymmetry we found qualitatively the same results: chicks from androgen-injected eggs showed less submissive behaviour (b = −2.110, s.e. = 0.5779, p<0.001), but submissive behaviour was not predicted by age (b = −0.0317, s.e. = 0.0328, p = 0.334), residual body mass (b = −1.589, s.e. = 1.323, p = 0.230), or bill length asymmetry (b = −0.030, s.e. = 0.230, p = 0.895).

### Dominance

**Figure 3:**
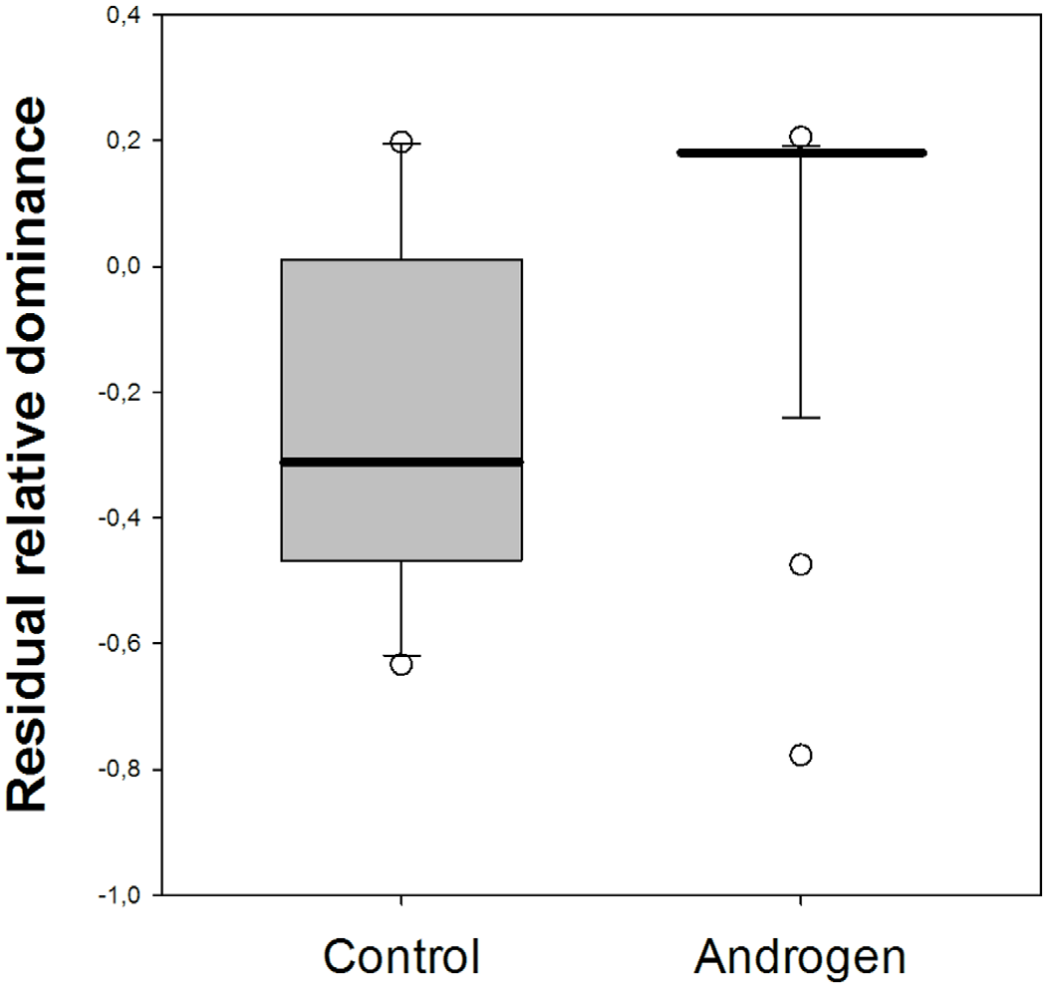
Residual relative dominance among dominant young in chicks coming from control and androgen-treated eggs. Residuals produced by linear mixed effects regressions containing individual as a random factor.

We calculated a dominance score for all chicks and then calculated the difference in dominance score between siblings within dyads containing opposite treatments, and identified the chick with the higher score as the dominant chick in the pairing. Out of the 37 dyads in which chicks showed some aggression or submission, in 10 dyads (distributed over 7 individuals) the control chick was dominant, and in 26 dyads (distributed over 19 individuals) the androgen chick was dominant, and in one dyad, chicks were equally dominant. We found that dominant androgen chicks had a higher dominance score than did dominant control chicks (b = 0.133, s.e. = 0.030, p = 0.0001, n = 36 dyads, 26 individuals). In a separate model we also found that dominant androgen chicks were relatively more dominant compared to their siblings than were control chicks (b = 0.362, s.e. = 0.094, p = 0.0005, n = 36; Fig. 3). We also found that previous dominance status did not significantly predict current dominance status (b = −0.0789, s.e. = 0.0701, p = 0.260, n = 36).

### Begging

Androgen chicks did not perform more frequent bouts of begging vocalizations than did control chicks (b = −0.159, s.e. = 0.455, p = 0.727, n = 386 observations, 31 pairings, 33 individuals, 102 total bouts of begging vocalizations). We also found no significant relationship between bouts of begging vocalizations and residual body mass (b = −0.0133, s.e. = 0.017, p = 0.428) or mass differences among siblings (b = 0.0192, s.e. = 0.0215, p = 0.372). In a second model, relative dominance showed no significant relationship with begging vocalisations (b = 0.144, s.e. = 0.705, p = 0.829), nor residual body mass (b = −0.018, s.e. = 0.018, p = 0.329).

In analyses of data from the standardized begging tests, frequency of begging pecks (b = −0.183, s.e. = 0.527, p = 0.729, n = 34) or begging thrusts (b = −0.182, s.e. = 0.430, p = 0.6720) were not affected by treatment (mean pecks by control chicks = 26.15, s.e. = 7.24; mean pecks by T chicks = 22.62, s.e. = 4.46; mean thrusts by control chicks = 11, s.e. = 2.75; mean thrusts by T chicks = 12.52, s.e. = 2.91). In a second set of models, we also found no effect of relative dominance on begging pecks (b = −0.210, s.e. = 0.631, p = 0.74) or begging thrusts (b = −0.284, s.e. = 0.468, p = 0.543).

### Competition for Food

**Figure 4:**
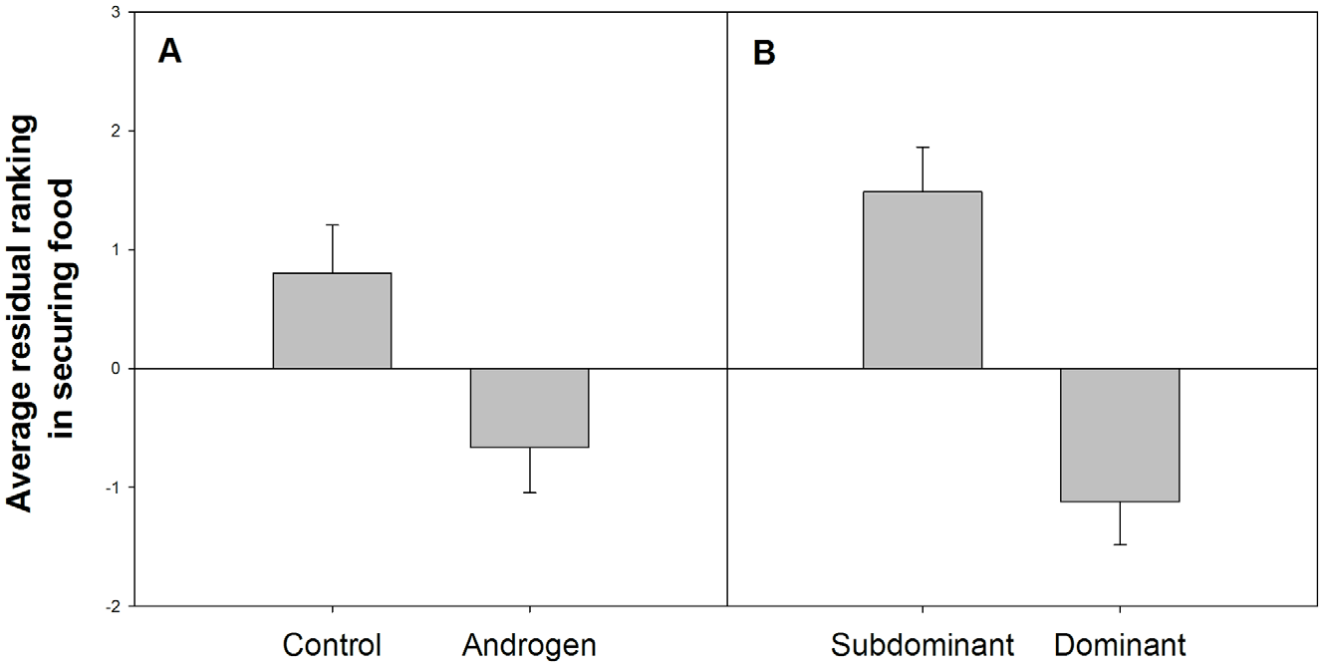
Residual ranking in competition for food in chicks coming from control and androgen-treated eggs (A) and in subdominant and dominant chicks (B). Ranking indicates the trial numbers (in a series of 10–15 trials) for which the focal chick succeeded in taking the food before its sibling. Therefore, a lower ranking value indicates more success in obtaining food in the earlier trials and a higher ranking indicates success in obtaining food only in the later trials. Residuals produced by linear mixed models containing cage as a random factor.

We found that within a competition test for food comprising several sequential trials, androgen chicks won more of the early trials and control chicks won more of the later trials (b = −1.465, s.e. = 0.562, p = 0.01, n = 137 trials across 13 dyads, Fig. 4a). Then we used the same model but replaced the fixed factor of treatment with relative dominance. We found that higher relative dominance predicted early access to food even more strongly than treatment (b = −0.058, s.e. = 0.012, p = 0.0001, Fig. 4b).

## Discussion

Mothers are suspected to bestow phenotypic advantages on particular offspring to facilitate efficient reduction of the number of offspring in case of insufficient food [6], [8]. Avian mothers systematically vary androgen concentrations over the laying sequence [14], , and the diversity in these patterns among species generated much interest in whether they correlate with particular reproductive strategies (e.g. hatching asynchrony adjustment hypothesis: [20]). For example, it has been suggested that a pattern of decreasing yolk androgens over the laying sequence of eggs within a clutch might support siblicide-mediated brood reduction as elevated yolk androgens may help the oldest dominant chick outcompete the younger ones [24]. We tested this for the first time by combining a comparative and experimental approach, using the facultatively siblicidal black-legged kittiwake. Our results do not support this hypothesis.

First, we found that yolk androgens did not decrease, but increased steeply, over the laying sequence (Fig. 1) as was reported in [25]. This pattern fits the idea that mothers compensate delayed hatching in marginal young by boosting their competitive ability (e.g. [20], [39]). Clearly, this does not fit the idea that siblicidal species like the kittiwake use yolk androgens to aid core young in eliminating marginal young, as has been implied for the cattle egret [24]. In kittiwakes, first-hatching core chicks inevitably assume the dominant position in the social hierarchy usually within 24 hours of the marginal chick’s hatching [30] so hatching position, and not yolk androgens, seem to determine dominance position within the brood. In fact, in all siblicidal species, dominance hierarchies always correlate perfectly with hatching sequence [19], whereas dominance hierarchies do not consistently correlate with a species’ pattern of yolk androgens over the laying sequence (decreasing pattern: cattle egret [24], blue-footed booby [26]; flat pattern: brown booby and blue-footed booby [27]; increasing pattern: blue-footed booby [26], black-legged kittiwake [25,this study], Australian pelican, G. Johnston, unpublished data). Clearly, the size and developmental advantage of core young supersedes any potential effects of yolk androgens in determining dominance during the early nestling period.

Given the observed pattern of increasing yolk androgens in the kittiwakes, one would expect that (1) similar to other species with increasing patterns, kittiwake mothers might allocate more maternal androgens to marginal young to increase their competitive ability to access resources, which may manifest itself in enhanced growth, begging, or scrambling for food; (2) elevated yolk androgens do not increase aggressive behaviour, otherwise they would counteract the establishment of a clear dominance rank order in the nest, undermine efficient siblicide in the event that siblicide occurs, and intensify aggressive interactions among the siblings. While we found some evidence for (1), our results clearly stand in contrast to (2).

With regard to (1), experimentally elevated androgens affected chick performance positively in scramble competition for food, but we found no effect of this treatment on growth. This may be due to the fact that apart from the sibling competition test, chicks were hand-fed individually and siblings did not have to compete for food. However, we also found no effect of treatment on the frequency with which androgen and control chicks performed bouts of begging vocalizations in their home cages. From a functional point of view, the latter finding might not be surprising as vocal begging cues have been suggested to be most important for determining the overall level of brood provisioning (although they sometimes also affect food allocation within broods, e.g. [40]). Forbes [41] suggested that if intensity of a brood’s vocal begging determines the amount of food delivery, then core siblings might actually benefit from vocal begging by marginal chicks [41], [42]. Furthermore, if begging vocalizations signal the hunger level of a chick honestly to parents, maternal androgens should not interfere with such essential offspring communication to parents. Indeed, several experimental studies have failed to find an effect of yolk androgen exposure on begging vocalizations [42], [44].

There was also a lack of a treatment effect on begging in the begging tests. This might be due to the fact that we tested the chicks alone, outside the context of sibling competition. Previous experimental studies demonstrated that yolk androgen exposure enhances bill gaping and vigor of begging shown by posture, as well as competitive ability [42], [45], [44], [23]. In general, such conspicuous visual displays are considered more important in determining within-brood allocation of food [46]–[49]. But these positive effects of yolk androgen exposure on actions involved in scramble competition manifest themselves mostly when chicks are tested in the presence of their siblings (e.g., canaries [23], European starlings [44], black-headed gulls [43]) but not when chicks are tested alone (canaries [50], European starlings [44], yellow-legged gulls [51], but see [45]). Indeed, when we measured begging effort via standardized solitary begging tests, we found no effect of androgen treatment on begging pecks or begging thrusts. But when chicks were presented with food in their home cages in the presence of their sibling, androgen chicks performed better than control chicks in securing the first meals. This finding supported the first expectation, and suggested that kittiwake mothers might deposit higher yolk androgens into second eggs to help marginal chicks compete for food.

Despite the increase of androgens over the laying sequence, experimentally elevated yolk androgens strongly increased aggression and dominance status, clearly in contrast with the second expectation. Also, control chicks showed much more frequent submissive behaviour in response to aggression than did androgen chicks. Core young in siblicidal species use aggression towards marginal siblings to secure dominant status [19] which gives them power to control food distribution within the brood. Indeed, social dominance in the sibling dyads turned out to be an even better predictor of who secured the first meals than androgen treatment. These findings create a problematic paradox: in a natural setting, early-hatching core chicks, which are exposed to lower yolk androgens, inevitably assume the dominant role due to their larger size and have the power to control food distribution and kill their subdominant siblings [52], [19], yet in our experiment eliminating hatching asynchrony, we found that the higher yolk androgen exposure characteristic of marginal chicks largely determines dominance. Why then do mothers allocate higher yolk androgens to marginal young if they can never reap the benefits for sibling competition due to their inferior social rank determined by delayed hatching?

We suggest an avenue by which benefits of higher yolk androgen exposure may manifest after marginal young become victims of siblicide. In poor years, marginal young are almost always ejected from the nest following an onslaught of violent attacks by the core sibling [29]. Kittiwakes live in large dense colonies situated on cliffs in which rows of nests sit along stratified horizontal ledges of rock [29]. Siblicidal attacks from the core offspring force marginal offspring out of the nest and into adjacent nests or into nests below [53]. Kittiwakes have high rates of adoption, at least 8% or higher [53]–[55], facilitated by kittiwake parents accepting foreign chicks of various ages in their nests [29], [53]. Vagrant chicks readily enter many foreign nests [53] and respond indiscriminately to any adult [56]. Ejected marginal chicks have been observed to enter nests in which the resident chicks are of similar or smaller size, expel resident chicks, and then survive in the foster nests until fledging [54], [53]. This fits very well with our finding that subdominant social status in the natal nest does not undermine a chick’s prospects of achieving dominance in a new nest containing a different chick. Higher maternal androgen exposure should increase their odds at winning such contests for dominance. An 8% adoption rate indicates that, even after siblicide, ejected marginal chicks retain substantial reproductive value and mothers stand to gain a large increment in fitness via adoption of their marginal chicks without incurring associated rearing costs.

Müller and Groothuis [39] suggested that mothers aiming for survival of the whole brood should produce an increasing pattern of yolk androgens which would promote the survival of marginal chicks in good years. We also show that flat or decreasing patterns of yolk androgens occur in species that produce marginal chicks primarily for insurance, in which marginal chicks merely act as replacement units for failed core eggs or chicks. According to this model, black-legged kittiwakes, which show a strong increase in yolk androgens over the laying sequence, should reap more reproductive value from survival of marginal young alongside the core (“extra” reproductive value [6]) compared to insurance value. Data from [30] indicate that marginal kittiwakes do contribute more extra reproductive value (0.37), than insurance value (0.045, see [6] for methods of calculation). In addition, we suggest that adoption represents a third component of their reproductive value that has thus far been overlooked. Adoption occurs at high rates in many other colonial seabirds as well (e.g., herring gull: 5–35% of pairs [57]; ring-billed gull: 4–38% of pairs [58]; common gull: 23.4% of pairs [59]; Audouin’s gull: 18.5–48% [60]; common tern: 15–24% of pairs [61]; Little tern: 29% of pairs [62]) but also in other birds (e.g. American avocet: 19.8–32.2% of pairs [63]; Greylag goose: up to 50% of pairs [64]) and at various stages of development (nest-switching at fledging, e.g. 5.4% of Eagle owlets [65], Eastern bluebird [66], [67]). Many of these avian families show increasing patterns of yolk androgens over the laying sequence (e.g. gulls [68]–[70], tern [71], owl [72], Eastern bluebird [73]), suggesting that higher yolk androgen exposure in marginal young may enhance the odds that mothers might profit from having a marginal offspring successfully reared in a foreign nest. We therefore propose the “adoption facilitation hypothesis” which states that mothers deposit relatively more yolk androgens into marginal eggs to increase the survival prospects of last-hatching young by enhancing their ability to appropriate parental care from unrelated adults.
